# Body Weight, Weight Self-Perception, Weight Teasing and Their Association with Health Behaviors among Chinese Adolescents—The Shanghai Youth Health Behavior Survey

**DOI:** 10.3390/nu14142931

**Published:** 2022-07-17

**Authors:** Yinliang Tan, Weiyi Lu, Wenxin Gu, Zhiping Yu, Jingfen Zhu

**Affiliations:** 1 School of Public Health, Shanghai Jiao Tong University, Shanghai 200025, China; melodybeata@sjtu.edu.cn (Y.T.); vivienlu999@shsmu.edu.cn (W.L.); guwenxin@sjtu.edu.cn (W.G.); 2 Department of Nutrition and Dietetics, University of North Florida, Jacksonville, FL 32224, USA; z.yu@unf.edu

**Keywords:** health risk behavior, body mass index, weight perception, weight teasing, adolescents, Chinese

## Abstract

Weight-related status has been associated with the physical and psychological health of adolescents. This cross-sectional study evaluated three different kinds of weight-related statuses (Body Mass Index (BMI), weight self-perception and weight teasing from others) among Chinese adolescents and identified their associations with health risk behaviors (lack of healthy dietary behavior, unhealthy dietary behavior, binge eating behavior, lack of physical activity (PA), sedentary behaviors (SB) and sleep disturbance). A stratified random cluster sampling method was used to select 10,070 students aged 11–18 years old from schools in Shanghai. Self-reported questionnaires were collected, weight-related statuses were divided into three categories and six specific health risk behaviors were classified into two groups: positive or negative. Overall, 27.82% of the adolescents were classified as being overweight and obese (35.61% of boys and 18.21% of girls), 43.45% perceived themselves as too heavy and 30.46% experienced weight teasing in the past. Among overweight or obese participants, 50.55% have been teased about their weight, and 77.48% perceived themselves as too heavy. Weight perception and weight teasing were significantly associated with health risk behaviors rather than the actual body weight status based on BMI, especially regarding binge eating behavior (body weight status (BMI): *p* > 0.05, underweight perception: OR = 1.18, 95%CI 1.03–1.34; weight teasing for more than once a year: OR = 2.00, 95%CI 1.76–2.27). In addition, weight perception and weight teasing were significantly associated with health risk behaviors, mainly in normal and overweight/obese groups but not in underweight groups. Weight teasing and weight self-perception play an independent and stronger role than actual body weight in the health behaviors of adolescents. This calls for more attention and intervention to reduce peer bullying and stigmas on weight among adolescents.

## 1. Introduction

Adolescence is a crucial period in developing a positive or negative body image [1], which could have a long-term impact on adult weight status and health [2,3]. Different types of weight-related statuses such as body weight measured by the body mass index (BMI), weight self-perception and weight teasing by others have been identified and studied in adolescent weight research [4,5]. Regarding weight status measured by BMI, nearly one-third of 12,016 adolescents in the United States were overweight and obese according to the national Youth Risk Behavior Survey data [6]. In China, the overweight and obesity prevalence among children and adolescents has been increasing steadily, escalating from 1% and 0.1% in 1985 (7–18 years old) to 11.1% and 7.9% in 2019 (6–17 years old) [7]. Related to but different from well-utilized BMI, weight self-perception is how an individual assesses his or her weight status [8], and weight teasing is a form of body shaming, which means that an individual is criticized for his or her body imperfection by others [9]. Both have been shown to be associated with obesity and health behaviors in adolescents [4,6,10]. In recent years, researchers reported that, regardless of actual body weight status (BMI), adolescents can think themselves as overweight/obese and suffer teasing from family or friends because of their weight [5,11]. More specifically, the rate of weight misperceptions was higher in girls (girls: 59.4%, boys: 40.6%) and in those who were overweight/obese (overweight/obese: 47.7% vs. underweight: 36.1% vs. normal weight: 36.5%) [4]. Similar results have been reported regarding weight teasing and stigmas. Weight stigmas were more prevalent in adolescents who were overweight/obese, and the gender differences remained mixed [12].

Related to weight problems, studies have reported that some health risk behaviors were more frequent in obese adolescents, such as less intake of fruits and vegetables, increased consumption of high caloric food, less physical exercise and poor sleep quality [13]. Similarly, adolescents with unmatched weight perceptions suffered from negative mental health [14,15] and unhealthy eating behaviors, such as skipping breakfast and higher soft drink and snack consumption [4,16]. Furthermore, in some research, weight perception could be more aggressively harmful than BMI on behaviors such as school absenteeism and suicide attempts [17,18]. Weight teasing, on the other hand, has been shown to have a strong association with disordered living habits, such as disordered eating in American adolescents [3]. Stigmas could also contribute to poor sleep quality, mediated by stress and depression in Chinese adolescents [19].

Although all types of weight-related statuses are important, prior studies mainly focused on a single weight-related status, especially body weight (BMI), when assessing the health status and well-being of adolescents. The research on the association among different types of weight-related statuses, as well as their relationships with health behaviors among adolescents, is still lacking. In this study, we explored three aspects of weight-related statuses, including body weight measured by BMI (objective), weight perception (self-subjective) and weight teasing from others (others-subjective), among adolescents in Shanghai city, where “leanness culture” is spread widely through fast-paced social communication and information flow. Their relationships with adolescent eating behaviors, physical activity, sedentary behavior and sleep disturbance were explored and compared as well. We hypothesized that weight perception and weight teasing may have a greater influence on health behaviors among adolescents than actual body weight.

## 2. Materials and Methods

### 2.1. Study Design and Sample

This is a cross-sectional study that was conducted in public schools as the 2021 part of a longitudinal cohort of the Youth Health Behavior Survey in Shanghai, China. The detailed sampling method is described in our previous report [20]. Briefly, the sample was selected using a stratified random cluster sampling method in May 2021. First, 3 districts were selected randomly from 16 districts in Shanghai. Second, schools in each district were stratified according to school types: junior high schools, senior high schools and vocational high schools. Eventually, 12 junior high schools (4367 students, aged 11–14 years old), 6 senior high schools (2682 students, aged 15–18 years old) and 3 vocational high schools (2145 students, aged 15–18 years old) were selected.

### 2.2. Data Collection

All students in the selected schools were invited to complete an online anonymous questionnaire in the school computer rooms. Teachers who had been priorly trained were present to explain the survey to students, to show them instructions and to answer any questions they had during this process. In total, 10,070 students were invited, among whom 876 students did not pass the quality check (i.e., they finished the questionnaire too quickly (<300 s)). In the end, 9194 students were included in the final analysis. This research was approved by the Ethics Committee of the School of Public Health, Shanghai Jiao Tong University (SJUPN-202016), and the informed consent of all participating students was obtained.

### 2.3. Measurements

The questionnaire was designed based on the Youth Risk Behavior Surveillance System (YRBSS) [21] in America and the Adolescent Health Related Behavior Survey of Shanghai Municipal Center for Disease Control and Prevention. Information regarding socio-demographic characteristics, the three kinds of weight-related statuses, dietary behaviors, physical activity, sedentary behavior and sleep disturbance was collected.

#### 2.3.1. Body Mass Index (BMI) and Body Weight Status

BMI was calculated based on self-reported weight and height. Participants were categorized into “Underweight”, “Normal” and “Overweight and obese” groups based on screening standards for the malnutrition, the status of being overweight and the obesity of school-age children and adolescents in China [22,23].

#### 2.3.2. Weight Perception

The self-perception of weight status was assessed by the question, “What do you think of your weight?” Responses were categorized into three groups: “Too light”, “Just right” (reference in logistic regression) and “Too heavy”.

#### 2.3.3. Weight Teasing

The question and classification criterion were designed based on previous studies [24]. Students were asked: “How often are you teased about your weight?” The original five responses were categorized into three levels. Specifically, a response of “less than once a year” remained as the “less than once a year” category. Responses of “a few times a year”, “a few times a month” and “at least once a week” were grouped together as “more than once a year”. Participants that did not experience teasing were labelled as “Never”. We asked this question and categorized them into three levels, considering that having experienced some teasing is not the same as having no teasing experience at all. The cut-off point of “more than once a year” was selected based on previous studies in which being teased a few times a year or more was classified as having experienced weight-related teasing and was correlated to negative physical and psychological outcomes [24].

#### 2.3.4. Healthy and Unhealthy Dietary Behavior

The responses to eight questions about the frequency of the food intake of fruit, vegetables, milk, breakfast, sugar sweetened beverages (SSB), dessert, fried food and fast food during the past 7 days were collected. The intake of fruit, vegetables, milk, SSB, dessert and fried food ≥1 time/day and having breakfast every day were coded as “1: high frequency” based on the recommendations from the Dietary Guidelines for Chinese Residents (2016) [25], the previous report in our lab [20] as well as the Scientific Report of Youth Risk Behavior Surveillance (United States, 2019) [26]. Having fast food ≥3 times in the past 7 days was coded as “1: high frequency”, and <3 times was coded as “0: low frequency”, considering that ≥3 times per week of fast food consumption was negatively associated with health outcomes in studies of Chinese children and adolescents [27,28]. Healthy dietary behavior was calculated by summing up the fruit, vegetables, milk and breakfast behavior and then by dividing into two categories (≥3 and <3) based on the median. The “≥3” was used as the reference group in the logistic regression model. Unhealthy dietary behavior was the sum of SSB, dessert, fried food and fast food behaviors. It was then grouped into two categories: 0 (reference in logistic regression) and ≥1, also based on the median.

#### 2.3.5. Binge Eating Behavior

The assessment of binge eating behavior was based on previous instruments [29,30]. Participants were asked whether they had these experiences in the past four weeks: “eating too much food (much more than a normal meal) in a short period of time” and “the feeling of being out of control, which means they could not stop eating or control how much they were eating”. Only those having both experiences were assessed as having engaged in binge eating behavior.

#### 2.3.6. Physical Activity

Participants were asked about their physical activities during the past 7 days, and those who did moderate- to vigorous-intensity physical activity for more than 60 min every day and vigorous-intensity activities for at least 3 days were considered to be “meeting standards” (reference in logistic regression) according to the World Health Organization (WHO) Guidelines on Physical Activity and Sedentary Behavior [31] and the Physical Activity Guidelines for Children and Adolescents in China [32]. The rest of the participants who did not meet the standard were grouped together and labeled as “not meeting standards”.

#### 2.3.7. Sedentary Behavior

The average sedentary time (not including the time for studying) per day during the past 7 days was self-reported and grouped into “<2 h/day” (reference in logistic regression) and “≥2 h/day” based on guidelines from the WHO [31] and China [32].

#### 2.3.8. Sleep Disturbance

Sleep disturbance was evaluated by asking “What do you think of your sleep quality during the past month?” The participants who chose “Good” or “Very good” were classified as the “Good” group (reference in logistic regression). The rest who chose “Bad” and “Very bad” were classified as the “Poor” group.

#### 2.3.9. Other Variables

Covariables included self-reported age, sex (male or female), registered residence (local or nonlocal), monthly pocket money (<¥200, ¥200–399, ¥400–599 and ≥¥600), socioeconomic status (low, middle and high), living on campus (yes or no) and academic performance in class (top 25%, middle and bottom 25%). Socioeconomic status was assessed according to the occupations and educational level of parents based on previous studies [33]. The occupations (9 types) and education levels (5 types) were coded based on a previous study [33]. Socioeconomic scores were calculated by dividing the sum of the coding of the father/mother’s occupation and the coding of the father/mother’s educational level by two. Higher scores indicate higher socio-economic statuses of adolescents. The total score was divided into 3 ordinal grades (low, middle and high).

### 2.4. Statistical Analysis

All statistical analyses were performed using IBM SPSS 24.0. Descriptive statistics including percentages (N(%)) and means with the standard deviation (SD) were used. Variance analysis or a chi-squared test was performed to compare the socio-demographic characteristics among different weight-related statuses, as well as to compare the weight perception/teasing level among different body weight statuses (BMI). Multiple logistic regression was used to analyze the effect of different weight-related statuses on health risk behaviors, and the reference groups are described above for the respective health behaviors. *p* < 0.05 was selected as the statistical significance level.

## 3. Results

### 3.1. Sociodemographic Characteristics among Different Weight-Related Statuses

Table 1 shows the sociodemographic characteristics of all the participants according to weight-related statuses. There were 5081 (55.26%) boys and 4113 (44.74%) girls, with a mean age of 15.01 years old. The percentage of abnormal body weight statuses (BMI) was much higher in boys (underweight: 10.21%, overweight/obese: 35.61%) than in girls (7.83%, 18.21%); in particular, the rate of being overweight/obese almost doubled in boys. For the whole sample, the rate of being overweight/obese reached 27.82%, but that of the too heavy perception (43.45%) was much higher, especially in girls (47.15%). Boys were more likely to report themselves as too light (boys 24.48%, girls 13.32%). The rate of students having ever been teased about their weight was 30.46%, for which the rate was a little higher in girls (33.07%) than in boys (28.36%). About half of the students were from junior high schools, and only 16.27% of them lived on campus. Students in vocational high schools had the highest level of overweight/obesity prevalence (30.91%), and senior high school students were more likely to perceive themselves as too heavy (51.30%) and to suffer from weight teasing (33.74%) than those of junior high schools and vocational high schools. The prevalence of having been teased about weight for more than once a year was higher in students not living on campus (17.03%) than those living on campus (14.71%), and local students were more likely to perceive themselves as too heavy (local 45.96%, nonlocal 38.01%). There was an equilibrium distribution in socioeconomic status, and a majority of the students had pocket money of less than 200 yuan per month. Students with lower academic performance were more likely to be overweight/obese, to perceive themselves as too heavy and to experience weight teasing.

### 3.2. Distributions of Weight Perception and Teasing among Different Body Weight Statuses (BMI)

As shown in Table 2, with normal BMI, 33.81% of the students reported themselves as too heavy, the rate of which doubled in girls (43.89%) than in boys (22.67%). By contrast, more boys reported themselves as too light (boys 26.37%, girls 10.09%), although the overweight/obesity rate was higher in boys than in girls. In addition, the proportion of students having ever experienced weight teasing was higher in the overweight or obese group (50.55%) than in the normal weight (23.45%) or underweight (17.72%) groups. In the normal or overweight/obese groups, the rate of ever being teased about weight was higher in girls (normal 29.19%, overweight/obese 58.08%) than in boys (normal 17.11%, overweight/obese 47.43%). In the underweight group, however, this rate was higher in boys (boys 21.58%, girls 11.49%).

### 3.3. Associations between Weight-Related Statuses and Health Risk Behaviors

As shown in Table 3, when not adjusted for the other two kinds of weight-related statuses in model 1, being overweight/obese had a weak positive association with the risk of binge eating behavior (OR = 1.17, 95%CI 1.05–1.30, *p* = 0.003) and sedentary behavior (OR = 1.13, 95%CI 1.02–1.24, *p* = 0.020). This effect, however, disappeared after adjusting for weight perception and teasing in model 2. In model 2, being overweight/obese had a weak protective association with unhealthy dietary behavior (*p* = 0.012). Weight perception and teasing played more significant roles in both model 1 and model 2 than BMI. Moreover, weight teasing had a stronger association with most health risk behaviors than perception, especially for binge eating behavior and sleep disturbance. As shown in model 2, the perception of being “too light” was related to a higher risk of negative dietary behaviors (lack of healthy dietary behavior: OR = 1.21, 95%CI 1.07–1.38, *p* = 0.002; unhealthy dietary behavior: OR = 1.24, 95%CI 1.09–1.41, *p* = 0.001; binge eating behavior: OR = 1.18, 95%CI 1.03–1.34, *p* = 0.015). Similarly, being teased more than once a year showed a stronger association with dietary risk behaviors (lack of healthy dietary behavior: OR = 1.28, 95%CI 1.13–1.46, *p* < 0.001; unhealthy dietary behavior: OR = 1.41,95%CI 1.24–1.61, *p* < 0.001; binge eating: OR = 2.00, 95%CI 1.76–2.27, *p* < 0.001). Similar phenomena were found in physical activity (perception: *p* > 0.05; teasing for less than once a year: OR = 1.49, 96%CI 1.23–1.81, *p* < 0.001) and sleep disturbance (perceiving too light: OR = 1.52, 95%CI 1.31–1.76; perceiving too heavy: OR = 1.39, 95%CI 1.22–1.58, *p* < 0.001; teasing for more than once a year: OR = 2.27, 95%CI 1.99–2.60, *p* < 0.001).

### 3.4. Weight Perception and Teasing Associated with Six Health Risk Behaviors by Body Weight Status (BMI)

**Figure 1:**
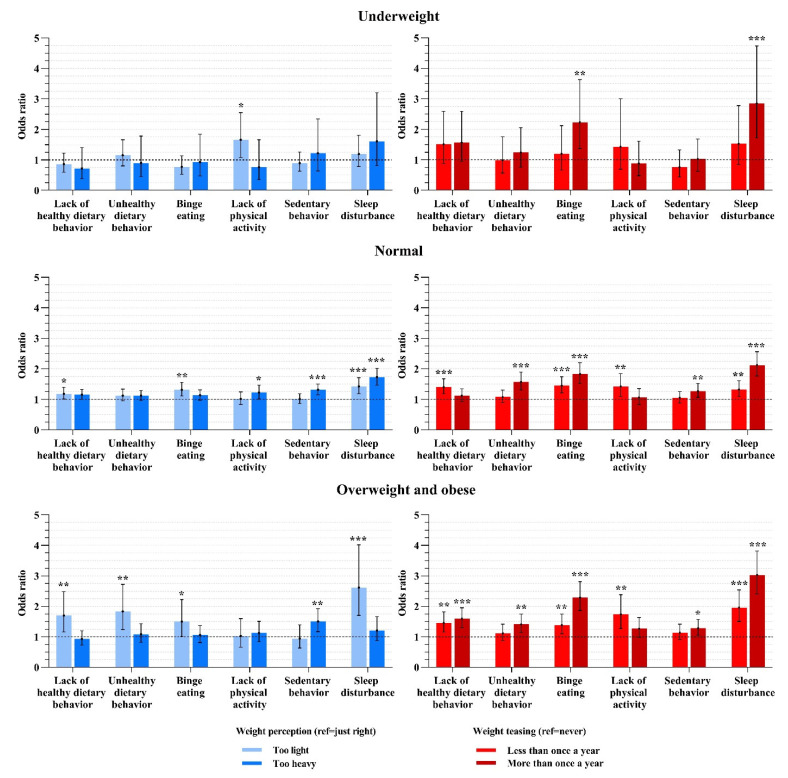
The association between weight perception or weight teasing and health risk behaviors according to different body weight status. Variables in the model were age, sex, registered residence, monthly pocket money, socioeconomic status, living on campus, academic performance in class, BMI, weight perception and weight teasing. * *p* < 0.05; ** *p* < 0.01; *** *p* < 0.001.

As shown in Figure 1, most of the significant associations between subjective weight-related statuses and health risk behaviors were in the normal and overweight/obese BMI groups. Regarding weight perception, the participants’ dietary risk behaviors were mainly correlated with perceiving themselves as “too light”, and those who perceived themselves as “too heavy” were more likely to sit for more than 2 h per day. Both these correlations were stronger in the overweight/obese group than in the normal weight group. Sleep disturbance was significantly associated with both kinds of weight perception. Physical activity only showed a weak correlation with weight perception. Regarding weight teasing, it was significantly associated with binge eating behavior and sleep disturbance in participants of any BMI group (normal, underweight, overweight and obese). Moreover, when the frequency of teasing rose, the odds of health risk behaviors rose. Less healthy dietary behaviors and physical activity were also related with teasing, and the odds were higher in the overweight/obese group. Unhealthy dietary and sedentary behaviors were only correlated with a high frequency of teasing without correlation intension changing among different body weight statuses (BMI).

## 4. Discussion

This study explored the statuses of body weight (BMI), weight self-perception, weight teasing from others and their relationships with health risk behaviors in adolescents of Shanghai, China. The results indicate that weight self-perception and weight teasing played a bigger influencing role than actual body weight in the health risk behaviors of adolescents, and weight teasing may have the strongest significant association with some behaviors, especially binge eating behavior and sleep disturbance.

We found that about one-third of students who were in the underweight and normal weight groups reported themselves as being too heavy, with a higher percentage in girls than in boys. Similarly, a previous US study reported that 27.1% of normal weight American adolescents perceived their weight as overweight [34]. This may be related to the “leanness and muscularity culture: the thinner, the better” that has been popular in society and social media [35,36]. The youth may be dissatisfied with their own weight or body shape, with girls pursuing leanness and with boys pursuing muscularity, in order to meet the body shape expectations of society, even if they actually have a normal or underweight status [37]. In addition, teasing others’ weight is another problem among young people, with studies showing that about a quarter to half of adolescents were bullied because of their weights [12]. In addition, weight stigmas seem to be more frequent in heavier groups and girls, as shown in our findings. This is consistent with previous reports, in which even though both boys and girls experience this kind of societal pressure, girls suffered more greatly [1,38].

The current study also showed that weight perception and teasing were associated with health risk behaviors, and objective body weight status (BMI) had little or no effect. Apart from that, weight teasing had a stronger impact than perception on some of these behaviors. The findings of these associations are similar to previous studies focusing on other health behaviors. For example, students with perceptions of abnormal body shape (either too heavy or too light) were at an increased risk of school absenteeism or even suicide-related behaviors, regardless of their BMI [17,18]. It was speculated that adolescents with abnormal weight perceptions or who were being teased suffered from negative mental effects, such as stress, low self-esteem, depression and body dissatisfaction [14,38,39], which are correlated with health risk behaviors among adolescents [40,41]. The reason for the stronger association between health risk behaviors and weight teasing than the association with weight perception in the current study may be due to weight teasing also reflecting stigmas/discrimination or school bulling, a behavior beyond the assessment of weight status [42]. Weight teasing may result in not only the feeling of low self-esteem, stress or depression, but also the feeling of being isolated from peers or family. Consequently, weight teasing may cause more serious effects on adolescents’ mental health than weight self-perception [8,43]. Moreover, teasing also weakens the protective effect on mental well-being from the social support of friends [44]. Therefore, as we work on helping teenagers to maintain a healthy weight, attention should also be paid to the occurrence of school bullying/victimization on weight, as well as the importance of weight-related messages in social media (both offline and online), especially among adolescents with heavier weights.

Regarding weight perception, the current study reported that greater dietary problems (less healthy diet choices, more unhealthy diet choices and more binge eating behaviors) were only related to self-perceiving as “too light” with respect to weights. This association was stronger among overweight/obese participants. This result may be explained by some previous studies. For example, one study showed that adolescents with underweight misperceptions may lack control on their diets (such as having more soft drinks and cookies instead of fruits and vegetables) [4]. In another study, students who thought they were heavy were more restrictive with their diet [45]. Different from the findings on dietary behaviors, sedentary behavior was positively associated with the perception of being “too heavy” in our study. According to our classification, sedentary behaviors refer to screen time, including the time spent on the TV, phone, internet or tablet. Thinness and slim models are often promoted on TV or social media [37,46]. Receiving too much of that information from screen media and various networking platforms (such as WeChat in China) [47,48] may make adolescents more likely to perceive themselves as being overweight and may pursue thinner body shapes.

Weight teasing was strongly correlated with binge eating behaviors and sleep disturbance in all weight statuses (especially the underweight or overweight/obese groups), and the relationships became stronger when the frequency of teasing rose. The strong association between teasing and those two health risk behaviors may be attributed to two aspects: first, teasing has a stronger effect on the psychological factors than weight perception and actual body weight (BMI) [8]; second, binge eating behavior and poor sleep quality may be influenced by bad mental health statuses, which are caused by weight teasing. For example, stigmas increase the level of stress and depression [19,49], which triggers eating and directly affects fat storage [50]. Based on this, interventions for food addiction and psychological distress may benefit adolescents who overeat due to weight stigmas [49]. Moreover, the association between teasing and binge eating behavior may also be attributed to teasing’s effect on adolescents’ weight control behaviors under the “leanness idealization” culture. When students are teased about their weights, they are usually also teased about their appearance and body parts, such as legs, arms, etc., resulting in their body dissatisfaction at the same time [43]. This kind of body dissatisfaction is not only associated with binge eating behavior directly but also with the adoption of unhealthy weight control behaviors in order to be thinner or more muscular, which may create a psycho-physiological disorder, leading to a rebound in binge eating [51]. Regarding sleep disturbance, since adolescents who experience weight teasing are mostly overweight or obese, their poor sleep quality may also be attributed to the lack of physical activity, excessive food intake before going to bed or a higher likelihood of snoring [52]. To overcome the negative effects of weight teasing/stigmas, it has been suggested that engaging in coping responses such as a healthy lifestyle and positive self-talk can be helpful [53].

This research has several strengths. We followed strict sampling methods and had a large sample size, covering the participants not only in junior and senior high schools, but also in vocational high schools. We categorized the weight-related statuses into three dimensions and found their different characteristics or strengths in their associations with health risk behaviors. A limitation of this study is that the study was only conducted in Shanghai, a modern urban area. The findings may or may not be the same in areas with lower economic status populations. This is a cross-sectional study which cannot verify causality. The adolescents’ height, weight and various kinds of health risk behaviors were self-reported instead of actually measured, which is subject to respondent bias. The frequency of food intake only referred to the past 7 days, which may not be the best representation of a participant’s habitual dietary patterns.

## 5. Conclusions

A significant proportion of adolescents overestimate their weights and have experienced weight teasing. The association between actual body weight status (BMI) and health risk behaviors was minimal, whereas weight self-perception and weight teasing from others had a stronger correlation. Weight teasing was strongly associated with most of the health risk behaviors examined, especially for sleep disturbance and binge eating behavior. The findings call for more attention and intervention to reduce peer bullying and stigmas on weight among adolescents.

## Figures and Tables

**Table 1:** Sociodemographic characteristics according to weight-related statuses *.

	**Body Mass Index (BMI)**				**Weight Perception**			
	**Under-Weight**	**Normal**	**Overweight/Obese**	***p*-Value**	**Too Light**	**Just Right**	**Too Heavy**	***p*-Value**
Age	15.15 ± 0.07	15.00 ± 0.03	14.98 ± 0.04	0.099	14.87 ± 0.05	14.76 ± 0.04	15.28 ± 0.03	<0.001
Sex				<0.001				<0.001
Male	519 (10.21)	2753 (54.18)	1809 (35.61)		1244 (24.48)	1781 (35.05)	2056 (40.47)	
Female	322 (7.83)	3042 (73.96)	749 (18.21)		548 (13.32)	1626 (39.53)	1939 (47.15)	
School type				<0.001				<0.001
Junior high school	374 (8.56)	2778 (63.62)	1215 (27.82)		897 (20.54)	1828 (41.86)	1642 (37.60)	
Senior high school	228 (8.50)	1774 (66.15)	680 (25.35)		454 (16.93)	852 (31.77)	1376 (51.30)	
Vocational high school	239 (11.14)	1243 (57.95)	663 (30.91)		441 (20.56)	727 (33.89)	977 (45.55)	
Registered residence				0.022				<0.001
Local	599 (9.51)	3912 (62.13)	1786 (28.36)		1185 (18.82)	2218 (35.22)	2894 (45.96)	
Nonlocal	242 (8.35)	1883 (65.00)	772 (26.65)		607 (20.95)	1189 (41.04)	1101 (38.01)	
Living on campus				0.190				0.004
Yes	125 (8.36)	973 (65.04)	398 (26.60)		325 (21.73)	503 (33.62)	668 (44.65)	
No	716 (9.30)	4822 (62.64)	2160 (28.06)		1467 (19.06)	2904 (37.72)	3327 (43.22)	
Socioeconomic status				0.003				0.139
Low	255 (9.49)	1627 (60.52)	806 (29.99)		554 (20.61)	963 (35.83)	1171 (43.56)	
Middle	288 (8.72)	2083 (63.06)	932 (28.22)		617 (18.68)	1218 (36.88)	1468 (44.44)	
High	298 (9.30)	2085 (65.10)	820 (25.60)		621 (19.39)	1226 (38.28)	1356 (42.33)	
Monthly pocket money (¥)				0.134				<0.001
<200	418 (8.70)	3010 (62.66)	1376 (28.64)		967 (20.12)	1820 (37.89)	2017 (41.99)	
200–399	165 (8.89)	1190 (64.16)	500 (26.95)		312 (16.81)	728 (39.25)	815 (43.94)	
400–599	98 (9.12)	679 (63.16)	298 (27.72)		200 (18.60)	369 (34.33)	506 (47.07)	
≥600	160 (10.96)	916 (62.74)	384 (26.30)		313 (21.44)	490 (33.56)	657 (45.00)	
Academic performance				<0.001				<0.001
Top 25%	293 (8.97)	2168 (66.36)	806 (24.67)		681 (20.84)	1301 (39.83)	1285 (39.33)	
Middle	391 (9.15)	2702 (63.22)	1181 (27.63)		795 (18.60)	1602 (37.48)	1877 (43.92)	
Bottom 25%	157 (9.50)	925 (55.96)	571 (34.54)		316 (19.12)	504 (30.49)	833 (50.39)	
Total	841 (9.15)	5795 (63.03)	2558 (27.82)		1792 (19.49)	3407 (37.06)	3995 (43.45)	
	**Weight Teasing**							**Total**
	**Never**		**Less than Once a Year**		**More than Once a Year**		***p*-Value**	
Age	14.87 ± 0.05		14.76 ± 0.04		15.28 ± 0.03		<0.001	15.01 ± 0.02
Sex							<0.001	
Male	3640 (71.64)		648 (12.75)		793 (15.61)			5081 (55.26)
Female	2753 (66.93)		622 (15.12)		738 (17.95)			4113 (44.74)
School type							<0.001	
Junior high school	3058 (70.03)		525 (12.02)		784 (17.95)			4367 (47.50)
Senior high school	1777 (66.26)		446 (16.63)		459 (17.11)			2682 (29.17)
Vocational high school	1558 (72.63)		299 (13.94)		288 (13.43)			2145 (23.33)
Registered residence							0.267	
Local	4350 (69.08)		893 (14.18)		1054 (16.74)			6297 (68.49)
Nonlocal	2043 (70.52)		377 (13.01)		477 (16.47)			2897 (31.51)
Living on campus							0.020	
Yes	1044 (69.78)		232 (15.51)		220 (14.71)			1496 (16.27)
No	5349 (69.49)		1038 (13.48)		1311 (17.03)			7698 (83.73)
Socioeconomic status							0.078	
Low	1897 (70.57)		381 (14.17)		410 (15.26)			2688 (29.24)
Middle	2268 (68.67)		475 (14.38)		560 (16.95)			3303 (35.92)
High	2228 (69.56)		414 (12.93)		561 (17.51)			3203 (34.84)
Monthly pocket money (¥)							0.337	
<200	3369 (70.13)		637 (13.26)		798 (16.61)			4804 (52.25)
200–399	1293 (69.71)		257 (13.85)		305 (16.44)			1855 (20.18)
400–599	716 (66.60)		172 (16.00)		187 (17.40)			1075 (11.69)
≥600	1015 (69.52)		204 (13.97)		241 (16.51)			1460 (15.88)
Academic performance							<0.001	
Top 25%	2324 (71.14)		430 (13.16)		513 (15.70)			3267 (35.53)
Middle	2996 (70.10)		603 (14.11)		675 (15.79)			4274 (46.49)
Bottom 25%	1073 (64.91)		237 (14.34)		343 (20.75)			1653 (17.98)
Total	6393 (69.54)		1270 (13.81)		1531 (16.65)			

* Data are presented with N(%) or mean ± standard deviation.

**Table 2:** Distributions of weight perception and weight teasing among different body weight statuses (BMI) in the whole sample and by sex *.

	Underweight	Normal	Overweight/Obese	χ^2^ Test	*p*-Value
All participants					
Weight perception				2980.53	<0.001
Too light	594 (70.63)	1033 (17.82)	165 (6.45)		
Just right	193 (22.95)	2803 (48.37)	411 (16.07)		
Too heavy	54 (6.42)	1959 (33.81)	1982 (77.48)		
Weight teasing				736.79	<0.001
Never	692 (82.28)	4436 (76.55)	1265 (49.45)		
Less than once a year	67 (7.97)	699 (12.06)	504 (19.70)		
More than once a year	82 (9.75)	660 (11.39)	789 (30.85)		
Male					
Weight perception				2296.17	<0.001
Too light	403 (77.65)	726 (26.37)	115 (6.36)		
Just right	95 (18.30)	1403 (50.96)	283 (15.64)		
Too heavy	21 (4.05)	624 (22.67)	1411 (78.00)		
Weight teasing				533.47	<0.001
Never	407 (78.42)	2282 (82.89)	951 (52.57)		
Less than once a year	50 (9.63)	257 (9.34)	341 (18.85)		
More than once a year	62 (11.95)	214 (7.77)	517 (28.58)		
Female					
Weight perception				928.79	<0.001
Too light	191 (59.32)	307 (10.09)	50 (6.68)		
Just right	98 (30.43)	1400 (46.02)	128 (17.09)		
Too heavy	33 (10.25)	1335 (43.89)	571 (76.23)		
Weight teasing				326.24	<0.001
Never	285 (88.51)	2154 (70.81)	314 (41.92)		
Less than once a year	17 (5.28)	442 (14.53)	163 (21.76)		
More than once a year	20 (6.21)	446 (14.66)	272 (36.32)		

* Data are presented with *n* (%).

**Table 3:** The relationship between three kinds of weight-related statuses and six health behaviors OR (95%CI).

	Lack of Healthy Dietary Behavior	Unhealthy Dietary Behavior	Binge Eating	Lack of Physical Activity	Sedentary Behavior	Sleep Disturbance
Model 1						
Body weight status (BMI)						
Normal	1	1	1	1	1	1
Underweight	1.06 (0.91–1.24)	1.13 (0.97–1.32)	0.91 (0.78–1.08)	0.92 (0.76–1.12)	0.98 (0.84–1.14)	1.04 (0.88–1.24)
Overweight/obese	1.00 (0.90–1.10)	0.92 (0.83–1.03)	1.17 (1.05–1.30)	0.99 (0.87–1.13)	1.13 (1.02–1.24)	1.10 (0.98–1.24)
Weight perception						
Just right	1	1	1	1	1	1
Too light	1.22 (1.08–1.37)	1.27 (1.12–1.45)	1.19 (1.05–1.35)	1.12 (0.96–1.31)	1.03 (0.91–1.16)	1.04 (0.88–1.24)
Too heavy	1.09 (0.99–1.20)	1.09 (0.98–1.21)	1.38 (1.24–1.53)	1.17 (1.03–1.32)	1.37 (1.25–1.51)	1.10 (0.98–1.24)
Weight teasing						
Never	1	1	1	1	1	1
Less than once a year	1.33 (1.17–1.51)	1.02 (0.89–1.16)	1.41 (1.24–1.61)	1.51 (1.26–1.82)	1.14 (1.01–1.29)	1.59 (1.38–1.84)
More than once a year	1.24 (1.10–1.39)	1.34 (1.19–1.51)	2.06 (1.83–2.32)	1.13 (0.97–1.32)	1.41 (1.25–1.58)	1.77 (1.57–1.98)
Model 2						
Body weight status (BMI)						
Normal	1	1	1	1	1	1
Underweight	0.97 (0.82–1.15)	1.03 (0.87–1.23)	0.86 (0.72–1.03)	0.88 (0.72–1.09)	1.06 (0.90–1.25)	0.95 (0.79–1.15)
Overweight/obese	0.94 (0.84–1.06)	0.86 (0.76–0.97)	0.96 (0.85–1.08)	0.91 (0.79–1.06)	0.94 (0.84–1.05)	0.80 (0.70–0.91)
Weight perception						
Just right	1	1	1	1	1	1
Too light	1.21 (1.07–1.38)	1.24 (1.09–1.41)	1.18 (1.03–1.34)	1.11 (0.95–1.29)	1.01 (0.90–1.15)	1.52 (1.31–1.76)
Too heavy	0.98 (0.88–1.09)	1.01 (0.90–1.13)	1.07 (0.95–1.21)	1.12 (0.97–1.29)	1.30 (1.17–1.45)	1.39 (1.22–1.58)
Weight teasing						
Never	1	1	1	1	1	1
Less than once a year	1.36 (1.19–1.55)	1.05 (0.92–1.21)	1.38 (1.21–1.59)	1.49 (1.23–1.81)	1.05 (0.92–1.19)	1.44 (1.24–1.66)
More than once a year	1.28 (1.13–1.46)	1.41 (1.24–1.61)	2.00 (1.76–2.27)	1.11 (0.94–1.31)	1.26 (1.11–1.42)	2.27 (1.99–2.60)

Model 1: Adjusted for age, sex, registered residence, monthly pocket money, socioeconomic status, status of living on campus and academic performance in class. Model 2: Variables in the model were age, sex, registered residence, monthly pocket money, socioeconomic status, the status of living on campus, academic performance in class, body weight status (BMI), weight perception and weight teasing.

## Data Availability

The data presented in this study are available on request from the corresponding author.

## References

[1] Voelker D.K., Reel J.J., Greenleaf C. (2015). Weight status and body image perceptions in adolescents: Current perspectives. Adolesc. Health Med. Ther..

[2] Szwimer E., Mougharbel F., Goldfield G.S., Alberga A.S. (2020). The Association Between Weight-Based Teasing from Peers and Family in Childhood and Depressive Symptoms in Childhood and Adulthood: A Systematic Review. Curr. Obes. Rep..

[3] Hooper L., Puhl R., Eisenberg M.E., Crow S., Neumark-Sztainer D. (2021). Weight teasing experienced during adolescence and young adulthood: Cross-sectional and longitudinal associations with disordered eating behaviors in an ethnically/racially and socioeconomically diverse sample. Int. J. Eat. Disord..

[4] San Martini M.C., de Assumpção D., Barros M.B.A., Barros Filho A.A., Mattei J. (2021). Weight self-perception in adolescents: Evidence from a population-based study. Public Health Nutr..

[5] Pont S.J., Puhl R., Cook S.R., Slusser W. (2017). Stigma Experienced by Children and Adolescents with Obesity. Pediatrics.

[6] Dues K., Kandiah J., Khubchandani J., Haroldson A. (2020). Adolescent Body Weight Perception: Association with Diet and Physical Activity Behaviors. J. Sch. Nurs..

[7] Pan X.F., Wang L., Pan A. (2021). Epidemiology and determinants of obesity in China. Lancet Diabetes Endocrinol..

[8] Patte K.A., Livermore M., Qian W., Leatherdale S.T. (2021). Do weight perception and bullying victimization account for links between weight status and mental health among adolescents?. BMC Public Health.

[9] Woodworth A., Schneider M. (2021). Critical Evaluation of the Case for Pausing California’s School-based Fitness Testing. Health Behav. Policy Rev..

[10] Puhl R.M., Wall M.M., Chen C., Bryn Austin S., Eisenberg M.E., Neumark-Sztainer D. (2017). Experiences of weight teasing in adolescence and weight-related outcomes in adulthood: A 15-year longitudinal study. Prev. Med..

[11] Lee K., Dale J., Guy A., Wolke D. (2018). Bullying and negative appearance feedback among adolescents: Is it objective or misperceived weight that matters?. J. Adolesc..

[12] Puhl R.M., Lessard L.M. (2020). Weight Stigma in Youth: Prevalence, Consequences, and Considerations for Clinical Practice. Curr. Obes. Rep..

[13] Rupp K., Friel C.P. (2022). Changes in Health Behaviors Associated with Weight Gain by Weight Classification During the COVID-19 Pandemic. Am. J. Health Promot..

[14] Lee K.H., Bong S.H., Kang D.H., Choi T.Y., Kim J.W. (2020). Association Between Weight Misperception and Some Mental Health-Related Characteristics in Korean Adolescents. Neuropsychiatr. Dis. Treat..

[15] Dietz W.H. (1998). Health consequences of obesity in youth: Childhood predictors of adult disease. Pediatrics.

[16] Yan H., Wu Y., Oniffrey T., Brinkley J., Zhang R., Zhang X., Wang Y., Chen G., Li R., Moore J.B. (2018). Body Weight Misperception and Its Association with Unhealthy Eating Behaviors among Adolescents in China. Int. J. Environ. Res. Public Health.

[17] Duncan D.T., Hansen A.R., Woo Baidal J., Lyn R., Hill A., Zhang J. (2017). Perceived not actual overweight is associated with excessive school absenteeism among U.S. adolescents. Obes. Res. Clin. Pract..

[18] Eaton D.K., Lowry R., Brener N.D., Galuska D.A., Crosby A.E. (2005). Associations of body mass index and perceived weight with suicide ideation and suicide attempts among US high school students. Arch. Pediatr. Adolesc. Med..

[19] Wang Z., Dang J., Zhang X., Moore J.B., Li R. (2021). Assessing the relationship between weight stigma, stress, depression, and sleep in Chinese adolescents. Qual. Life Res..

[20] Zhu J., Tan Y., Lu W., He Y., Yu Z. (2021). Current Assessment of Weight, Dietary and Physical Activity Behaviors among Middle and High School Students in Shanghai, China-A 2019 Cross-Sectional Study. Nutrients.

[21] Centers for Disease Control and Prevention 2021 State and Local Youth Risk Behavior Survey. https://www.cdc.gov/healthyyouth/data/yrbs/pdf/2021/2021-yrbs-standard-hs-questionnaire.pdf.

[22] (2014). Screening Standard for Malnutrition of School-Age Children and Adolescents.

[23] (2018). Screening for Overweight and Obesity among School-Age Children and Adolescents.

[24] Haines J., Hannan P.J., van den Berg P., Eisenberg M.E., Neumark-Sztainer D. (2013). Weight-related teasing from adolescence to young adulthood: Longitudinal and secular trends between 1999 and 2010. Obesity.

[25] (2016). Dietary Guidelines for Chinese Residents. http://dg.cnsoc.org/article/2016b.html.

[26] Merlo C.L., Jones S.E., Michael S.L., Chen T.J., Sliwa S.A., Lee S.H., Brener N.D., Lee S.M., Park S. (2020). Dietary and Physical Activity Behaviors Among High School Students—Youth Risk Behavior Survey, United States, 2019. MMWR Suppl..

[27] Zhao Y., Wang L., Xue H., Wang H., Wang Y. (2017). Fast food consumption and its associations with obesity and hypertension among children: Results from the baseline data of the Childhood Obesity Study in China Mega-cities. BMC Public Health.

[28] Shan X.Y., Xi B., Cheng H., Hou D.Q., Wang Y., Mi J. (2010). Prevalence and behavioral risk factors of overweight and obesity among children aged 2-18 in Beijing, China. Int. J. Pediatr. Obes..

[29] Yanovski S.Z. (1993). Binge eating disorder: Current knowledge and future directions. Obes. Res..

[30] Johnson W.G., Grieve F.G., Adams C.D., Sandy J. (1999). Measuring binge eating in adolescents: Adolescent and parent versions of the questionnaire of eating and weight patterns. Int. J. Eat. Disord..

[31] WHO WHO Guidelines on Physical Activity and Sedentary Behavior. https://www.who.int/publications/i/item/9789240015128.

[32] Zhang Y., Ma S., Chen C., Liu S., Zhang C., Cao Z., Jiang F. (2017). Physical Activity Guidelines for Children and Adolescents of China. Chin. J. Evid. Based Pediatr..

[33] Zang F. (2017). The Influence of Stress and Depression on Smoking Behavior of Adolescents with Different Social Classes. Master’s Thesis.

[34] Mbogori T., Arthur T.M. (2021). Perception of Body Weight Status Is Associated with the Health and Food Intake Behaviors of Adolescents in the United States. Am. J. Lifestyle Med..

[35] Lavender J.M., Brown T.A., Murray S.B. (2017). Men, Muscles, and Eating Disorders: An Overview of Traditional and Muscularity-Oriented Disordered Eating. Curr. Psychiatry Rep..

[36] Wiseman C.V., Gunning F.M., Gray J.J. (1993). Increasing pressure to be thin: 19 years of diet products in television commercials. Eat. Disord..

[37] Solmi F., Sharpe Ph D.H., Gage S.H., Maddock J., Lewis G., Patalay P. (2021). Changes in the Prevalence and Correlates of Weight-Control Behaviors and Weight Perception in Adolescents in the UK, 1986–2015. JAMA Pediatr..

[38] Tang J., Yu Y., Du Y., Ma Y., Zhu H., Liu Z. (2010). Association between actual weight status, perceived weight and depressive, anxious symptoms in Chinese adolescents: A cross-sectional study. BMC Public Health.

[39] Page R.M., Allen O. (1995). Adolescent perceptions of body weight and weight satisfaction. Percept. Mot. Ski..

[40] Liu W., Cai T., Zhu H., Lu Y., Ling Y. (2016). The relationship between depression, anxiety, stress and adolescent emotionaleating: The mediating role of self-control. Chin. J. Clin. Psychol..

[41] Alipour B., Abbasalizad Farhangi M., Dehghan P., Alipour M. (2015). Body image perception and its association with body mass index and nutrient intakes among female college students aged 18–35 years from Tabriz, Iran. Eat. Weight. Disord..

[42] Gmeiner M.S., Warschburger P. (2021). Interrelation between weight and weight stigma in youth: Is there evidence for an obesogenic vicious cycle?. Eur. Child Adolesc. Psychiatry.

[43] Day S., Bussey K., Trompeter N., Mitchison D. (2021). The Impact of Teasing and Bullying Victimization on Disordered Eating and Body Image Disturbance Among Adolescents: A Systematic Review. Trauma Violence Abus..

[44] Ringdal R., Bjørnsen H.N., Espnes G.A., Bradley Eilertsen M.E., Moksnes U.K. (2021). Bullying, social support and adolescents’ mental health: Results from a follow-up study. Scand. J. Public Health.

[45] Song L., Zhang Y., Chen T., Maitusong P., Lian X. (2022). Association of body perception and dietary weight management behaviours among children and adolescents aged 6–17 years in China: Cross-sectional study using CHNS (2015). BMC Public Health.

[46] Selensky J.C., Carels R.A. (2021). Weight stigma and media: An examination of the effect of advertising campaigns on weight bias, internalized weight bias, self-esteem, body image, and affect. Body Image.

[47] Tiggemann M., Hayden S., Brown Z., Veldhuis J. (2018). The effect of Instagram “likes” on women’s social comparison and body dissatisfaction. Body Image.

[48] Brown Z., Tiggemann M. (2016). Attractive celebrity and peer images on Instagram: Effect on women’s mood and body image. Body Image.

[49] Ahorsu D.K., Lin C.Y., Imani V., Griffiths M.D., Su J.A., Latner J.D., Marshall R.D., Pakpour A.H. (2020). A prospective study on the link between weight-related self-stigma and binge eating: Role of food addiction and psychological distress. Int. J. Eat. Disord..

[50] Tomiyama A.J. (2014). Weight stigma is stressful. A review of evidence for the Cyclic Obesity/Weight-Based Stigma model. Appetite.

[51] Neumark-Sztainer D.R., Wall M.M., Haines J.I., Story M.T., Sherwood N.E., van den Berg P.A. (2007). Shared Risk and Protective Factors for Overweight and Disordered Eating in Adolescents. Am. J. Prev. Med..

[52] Lin C.Y., Imani V., Broström A., Huus K., Björk M., Hodges E.A., Pakpour A.H. (2020). Psychological distress and quality of life in Iranian adolescents with overweight/obesity: Mediating roles of weight bias internalization and insomnia. Eat. Weight. Disord..

[53] Himmelstein M.S., Puhl R.M., Quinn D.M. (2018). Weight stigma and health: The mediating role of coping responses. Health Psychol..

